# Connecting the dots between breast cancer, obesity and alcohol consumption in middle-aged women: ecological and case control studies

**DOI:** 10.1186/s12889-018-5357-1

**Published:** 2018-04-06

**Authors:** E. R. Miller, C. Wilson, J. Chapman, I. Flight, A.-M. Nguyen, C. Fletcher, Ij Ramsey

**Affiliations:** 1 College of Medicine and Public Health, Flinders University, Adelaide, Australia; 2 Flinders Centre for Innovation in Cancer, Flinders University , Adelaide, Australia; 3 Cancer Council of South Australia, Adelaide, Australia

**Keywords:** Breast cancer, alcohol, Obesity, Stress

## Abstract

**Background:**

Breast cancer (BC) incidence in Australian women aged 45 to 64 years (‘middle-aged’) has tripled in the past 50 years, along with increasing alcohol consumption and obesity in middle-age women. Alcohol and obesity have been individually associated with BC but little is known about how these factors might interact. Chronic psychological stress has been associated with, but not causally linked to, BC. Here, alcohol could represent the ‘missing link’ – reflecting self-medication. Using an exploratory cross-sectional design, we investigated inter-correlations of alcohol intake and overweight/obesity and their association with BC incidence in middle-aged women. We also explored the role of stress and various lifestyle factors in these relationships.

**Methods:**

We analysed population data on BC incidence, alcohol consumption, overweight/obesity, and psychological stress. A case control study was conducted using an online survey. Cases (*n* = 80) were diagnosed with BC and controls (*n* = 235) were women in the same age range with no BC history. Participants reported lifestyle data (including alcohol consumption, weight history) over consecutive 10-year life periods. Data were analysed using a range of bivariate and multivariate techniques including correlation matrices, multivariate binomial regressions and multilevel logistic regression.

**Results:**

Ecological inter-correlations were found between BC and alcohol consumption and between BC and obesity but not between other variables in the matrix. Strong pairwise correlations were found between stress and alcohol and between stress and obesity.

BMI tended to be higher in cases relative to controls across reported life history. Alcohol consumption was not associated with case-control status. Few correlations were found between lifestyle factors and stress, although smoking and alcohol consumption were correlated in some periods. Obesity occurring during the ages of 31 to 40 years emerged as an independent predictor of BC (OR 3.5 95% CI: 1.3–9.4).

**Conclusions:**

This study provides ecological evidence correlating obesity and alcohol consumption with BC incidence. Case-control findings suggest lifetime BMI may be important with particular risk associated with obesity prior to 40 years of age. Stress was ecologically linked to alcohol and obesity but not to BC incidence and was differentially correlated with alcohol and smoking among cases and controls. Our findings support prevention efforts targeting weight in women below 40 years of age and, potentially, lifelong alcohol consumption to reduce BC risk in middle-aged women.

## Background

The incidence of breast cancer (BC) in Australian women aged 45 to 64 years (‘middle-aged’) is reported to have tripled in the past five decades [1]. Middle-aged women are at particular risk of developing BC, compared to younger women, because of accumulating exposure to cancer risk factors [2]. While genetic and hormonal factors are recognised as important risk factors for BC, there is a range of lifestyle choices and personal factors (such as stress response) that may also contribute to BC risk. Identified lifestyle linked risk factors include alcohol consumption and being overweight or obese [3]. These risk factors have been primarily identified through correlational findings with BC incidence linked to the prevalence of excess alcohol consumption and obesity, particularly in the ‘at-risk’ cohort of middle-aged women [4–6].

Middle-aged women in developed countries, particularly those aged 45–59 years, consume alcohol more frequently than any other female age cohort [4, 6, 7] although the trend towards increasing patterns of alcohol consumption in later life is likely to be established in early adulthood [8]. Alcohol is classified as a Group 1 carcinogen [9], and has been strongly linked to many common cancers, including BC [10–12]. Epidemiological evidence describes a dose-response relationship between alcohol and BC although regular alcohol consumption, even at relatively low levels, may also increase the risk [1, 10, 13]. It has been proposed that oestrogen levels, elevated in women who consume alcohol, may be an important mechanism of BC development [13, 14].

The association between obesity and BC is also well established, although the mechanism by which obesity increases risk of BC is not fully understood. Nearly 28% of Australian females are now described as obese, with prevalence in 2013 peaking at 33% of women in middle-aged groups [5]. The most commonly proposed mechanism linking obesity to BC risk is through high serum levels of oestradiol (an oestrogen hormone), which known to be increased in obesity [15, 16]. Chronic inflammation, due to the action of secretory molecules associated with adipose tissue, has also been proposed as a biological pathway to BC [17]. Furthermore, evidence suggests that being overweight or obese increases the risk for post-menopausal (middle-aged) rather than pre-menopausal women [18], although long term weight gain during the pre-menopausal adult years may also increase BC risk later in life [15].

Further complicating this picture is a potential link between alcohol consumption and obesity. Alcoholic drinks can be considered ‘empty’ calories because they have no significant nutritional value and are additional to dietary energy intake. A standard drink in Australia contains 10 g of pure alcohol, equating to approximately 70 cal in energy [19], without considering other substances in the beverage, such as complex carbohydrates, which provide additional calories [20]. Therefore, increased energy intake through alcohol consumption can promote an energy imbalance, where intake exceeds output, and ultimately contribute to weight gain [21]. To date, however, there is only equivocal epidemiological evidence for a direct link between alcohol consumption and body weight [22, 23]. Some studies have identified a link between alcohol consumption and an increased risk of obesity/overweight [24–27], whereas others have found no link or an inverse relationship [28, 29]. Other studies have demonstrated a higher risk of weight gain and abdominal obesity in men who consume alcohol but an inverse relationship between obesity and heavy drinking in women [24, 26, 30, 31]. It is likely that the picture is complex with modifying factors such as quantity and patterns of alcohol consumption, individual genetic variability and lifestyle factors playing important roles [22, 23].

In addition to, or irrespective of, potential synergies between alcohol and weight gain, it is possible that obesity and alcohol consumption may represent ‘missing links’ between other personal and lifestyle factors associated with, but not directly linked to, BC development. Many women attribute their BC to stress [32–34] and some research suggest that self-reported psychological stress may be associated with increased BC risk [35]. Stressful life events were independently associated with BC in a large cohort of Finnish women followed for 15 years [36]. Findings such as these suggest that psychological stress resulting from major life events may play an important role in the development of BC. Psychological stress may contribute to obesity and alcohol consumption because over eating has been identified as a response to chronic stress [37] and, further, a positive feedback loop has also been proposed between obesity and stress [38]. Similarly, there is evidence to suggest that chronic psychological stress is an important influential factor in forming harmful patterns of alcohol consumption [39].

While alcohol and obesity have been *independently* investigated as BC risk factors, no published studies to date have examined the interaction *between* these and other risk factors for BC. Using an exploratory cross-sectional design, the primary objective of this study was to untangle the roles of alcohol and overweight and obesity as predictors of BC development in Australian women aged 45 to 64 years. A secondary objective was to investigate the potentially mediating role of alcohol and obesity in observed associations between psychological stress and BC.

## Methods

We conducted an ecological analysis to identify the link between BC incidence and the risk factors alcohol consumption, overweight and obesity and stress. This form of analysis utilises existing data to link average outcomes between risks and outcomes. We utilised publicly available, routinely collected data on BC incidence in Australian women aged 45 to 64 years, population alcohol consumption, overweight and obesity prevalence, and data on psychological stress prevalence. We also conducted a case control survey study of BC survivors diagnosed in their middle-ages (45 to 64 years) to investigate lifetime exposures to alcohol, overweight and obesity, and psychological stress. The study was approved by the Flinders University Social and Behavioural Research Ethics Committee.

### Ecological data management and analysis

Population data on BC incidence, alcohol consumption, overweight and obesity and psychological distress were obtained from a number of Australia-wide surveys. Based on the years for which other data were also available, BC incidence data for the years 1982–2011 were collected from the Australian Cancer Incidence and Mortality (ACIM) book for BC provided by the Australian Institute of Health and Welfare [40]. Incidence rates of BC in Australian women aged between 45 and 64 years, ‘middle-aged’, were used in the analysis.

Alcohol consumption data were provided by the Risk Factor Prevalence Study for the years 1980, 1983, and 1989 [41–43], and the National Drug Strategy Household Survey for the years 1995, 1998, 2001, 2004, 2007, 2010, and 2013 [44–50]. Alcohol consumption per 1000 population was calculated from the proportion of women aged 18–64 years who reported any regular alcohol consumption because any regular alcohol consumption may be associated with increased risk of BC [1, 10, 13]. The age range of 18 to 64 years was selected to reflect life-time exposure to alcohol rather than consumption, which may or may not be associated with concomitant BC incidence.

Overweight and obesity data in the form of measured body mass index (BMI) for the years 1995, 2007–2008, and 2011–12 were obtained from the National Health Survey [4, 51]. We collected self-reported weight and height data (used to estimate overweight and obesity and to calculate BMI) for the years 1989–1990, 2001, and 2004–2005 from the National HealthSurvey [4, 52]. A combination of objectively measured and self-reported BMI was used because both measures were not provided for all years. As with alcohol consumption figures, data relating to overweight and obesity in women aged 18–64 years were used in the analysis to reflect long term exposure. Overweight was defined as BMI 25–29.9 and obesity as BMI ≥30 [53].

Rates of psychological stress in females aged 18–64 years were collected from the National Health Survey for the years 2001, 2004–2005, and 2007–2008 [4, 54, 55], and the Australian Health Survey for the years 2011–2012 [51]. Psychological stress was measured in both survey instruments by the Kessler Psychological Distress Scale (K10) [56]. Data relating to high and very high psychological distress level were used in the analysis.

For each exposure variable, rates per 1000 Australian women were calculated and age-standardised using the 2011 population as the reference [57].

### Case-control study

A survey was conducted between mid-July and mid-August 2015. It involved 315 South Australian women aged 45 to 70 years. Cases (*n* = 80) were women first diagnosed with BC within the previous 5 years, when between the ages of 45 and 65 years, and controls (*n* = 235) were women in the same age range with no BC history. Participants were excluded if they had been diagnosed with any other cancer in the past, with the exception of non-melanoma skin cancer, which is very common in Australia but not considered life threatening. Cases and controls were recruited using a variety of methods including advertisements in community and commercial newspapers, organisational electronic newsletters and ‘word of mouth’. Cases were also recruited via BC support services. We calculated that we would need to recruit 50 cases and 104 controls to be able to identify a 20% exposure difference (equating to a relative risk of 2 based on an average of historical exposure prevalences [58]) with a power of 0.80 at the 0.05 significance level.

All participants completed an anonymous online questionnaire (prepared using Qualtrics, Provo, UT, July 2015). Full details about the study were provided on the survey landing page and, using the approach consistent with the Australian National Statement on Ethical Conduct of Human Research [59], participants were required to acknowledge their consent before proceeding. The survey consisted of questions relating to the participant’s lifetime alcohol consumption, weight history, psychological stress, language spoken and demographic information (specifically; age, country of birth and Australian Indigenous status). For the current analyses, age is the only relevant factor. Participants provided data on prior alcohol consumption, weight, stress, activity level and smoking during 10-year periods of their adult life – from 18 years to their age at time data collection.

#### Alcohol consumption

The Lifetime Drinking History Questionnaire (LDH-Q) [60] was modified to measure alcohol consumption over consecutive 10-year periods of the participants’ life and also included a measure of smoking status. Participants provided information regarding the number of days alcohol was consumed per month, the average and maximum number of drinks consumed per day, and the style of alcohol consumption (occasional, weekend, binge, or frequent (‘regular or everyday drinking’).

#### Weight and activity history

Following the method of Vasunilashorn et al. [61], participants reported their height and then their approximate weight during 10-year periods of their adult life. BMI was calculated for each 10-year period by dividing self-reported weight (kg) by current height-squared (m^2^). Approximate activity level during each 10-year period was also reported. Participants were provided a definition of all categories of activity, which were based on National Heart Foundation of Australia guidelines [62]. Participants selected from the options: ‘mainly light activity’ (defined as “little or no regular exercise beyond daily activities on most days”); ‘mainly moderate activity’ (“about 30 minutes of exercise at least 5 days a week”); ‘mainly high activity’ (“vigorous and sustained exercise nearly every day”); or ‘my physical activity level varied considerably throughout this period’. In most of our analyses, these categories were dichotomised to “mainly light activity” and “more than light activity”.

#### Psychological stress

An adapted version of the List of Threatening Experiences questionnaire (LTE-Q) [63] was included as a measure of exposure to stressful life events and was modified to include open ended questions about these events. The LTE-Q consists of 12 categories of common negative life events that are likely to be regarded as threatening. These include events such as being fired from a job, having a major financial crisis, and suffering from serious illness, injury, or assault. Participants were asked to identify any life events that had affected them during each 10-year period of their life.

### Data analysis

All data were analysed using Stata (release 15, Stata Corporation, College Station, TX, USA). Missing data points in the ecological analysis were interpolated by averaging differences between existing data points. This method of interpolation is used widely (for example see Geliebter et al. 2015 [64]) but can overly smooth peaks in the data and can therefore lead to less reliable estimates. Nonetheless, as stated by Çokluk and Kayri [65]; “If the researcher does not have other information, average value imputation is the best way of estimation” (page 304). Correlation coefficients were calculated for BC and each exposure variable individually and as a correlation matrix. Case control data were analysed descriptively for sample characteristics, and bivariately to assess differences between cases and controls across all time periods. To assess patterns reflective of differential recall, a comparison of response completeness from cases and controls was undertaken and demonstrated no difference in response patterns. Correlation matrices of each exposure variable were developed for cases and controls for each age period. Collinearity was assessed using Chi-Square and Phi statistics. Multivariate binomial regressions were then undertaken to identify independent predictor variables for case status at each age period. Finally, multilevel logistic regression was performed. Categorical by categorical interaction for obesity and alcohol in logistic regression was conducted for each of the life periods separately and as an interaction term within the multilevel logistic regression. Multi-collinearity was assessed by Eigensystem analysis of correlation matrix. All data were analysed at the 0.05 significance level.

## Results

### Ecological study

**Fig. 1:**
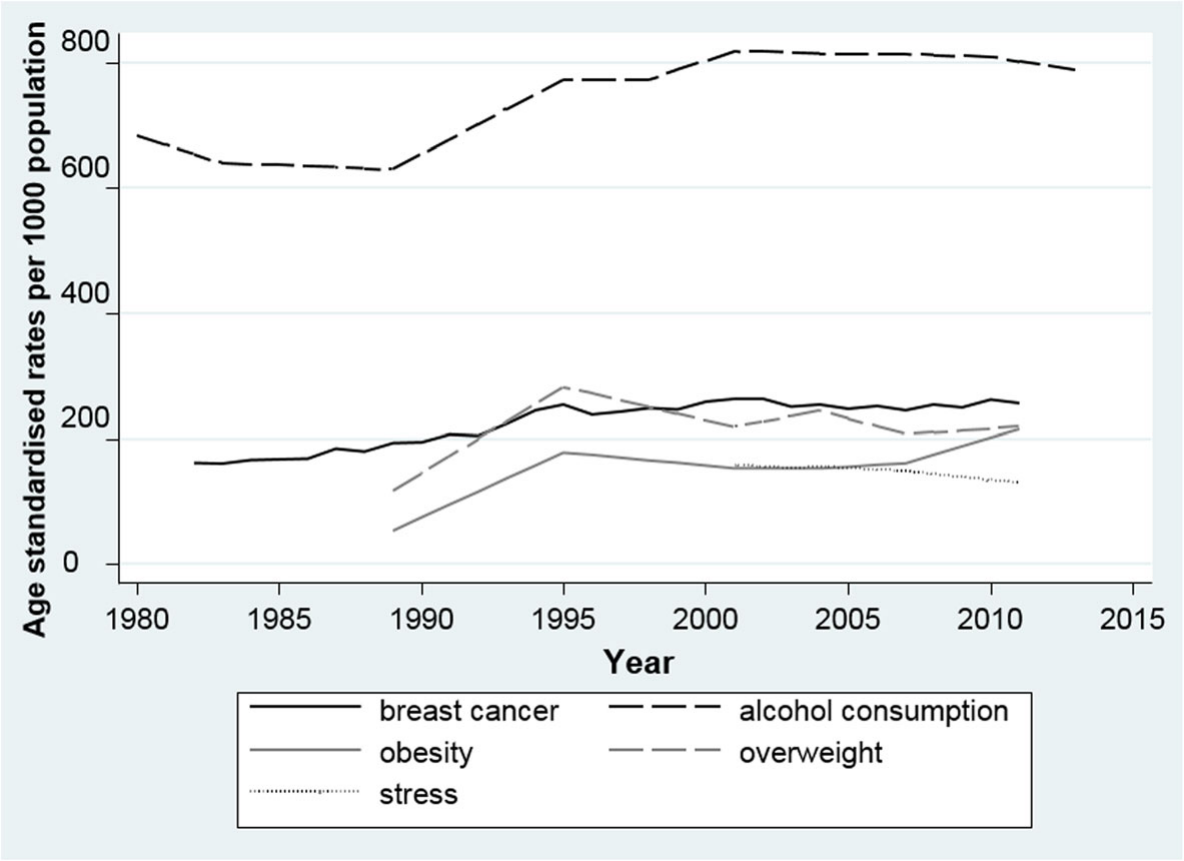
Incidence rates of breast cancer and rates of alcohol consumption, obesity, overweight and stress in Australian women 1980 to 2011. Note. BC incidence in women aged 45 to 64 years (rate × 100); any regular alcohol consumption in women aged 18 to 64 year; obesity and overweight as indicated by BMI in women aged 18 to 64 years; high and very high stress as measured by the Kessler Psychological Distress Scale

The age-standardised incidence rate of BC and rates of alcohol consumption, overweight, obesity and stress are plotted over time in Fig. 1. Along with increasing incidence of BC, rates of alcohol consumption and obesity have also increased over time. Steeply increasing rates of overweight occurred until 1995 but have been declining since then. Although population stress data are unavailable prior to 2001, there appears to be a decreasing trend for high to very high stress.

A strong positive correlation was found between BC incidence and alcohol consumption, as well as a moderate correlation between BC and obesity (see Table 1). However, no further significant inter-correlations were identified. Stress was omitted from the matrix due to small data numbers but a strong positive pairwise correlation was found between stress and alcohol consumption (Spearman’s rho = 0.90, *p* < 0.001) and a strong negative correlation between stress and obesity (Spearman’s rho = − 0.90, p < 0.001).

**Table 1:** Spearman’s rank correlation matrix for breast cancer incidence and rates of alcohol consumption, obesity and overweight*

	Breast cancer	Alcohol	Obesity	Overweight
Breast cancer incidence	1.00			
Alcohol consumption	0.78**	1.00		
Obesity	0.46***	0.23	1.00	
Overweight	0.23	0.17	0.37	1.00

*Stress not included due to insufficient data points

**p < 0.001

***p = 0.029

### Case-control study

We exceeded the number of cases and controls previously calculated as necessary to meet our power calculations. Eighty cases and 235 controls completed the survey and selected characteristics of the two groups are presented in Table 2. Consistent with their cancer status, cases reported significantly poorer current health status than did controls but there were few other differences reported at the time of completing the survey. The median age was approximately 57 years and 75% were born in Australia. Only one participant identified as Indigenous Australian (data not shown). The proportion of participants classifiable as obese (BMI > 30) tended to increase with each life period up until 61 years. Nearly all of the participants reported having consumed alcohol at some time, with a median age at first consumption of 16 years. Most (*n* = 192, 72%) had commenced regular consumption of alcohol before the age of 30 years; however a greater proportion of controls reported commencing regular drinking in the youngest age group (12 to 17 years).

**Table 2:** Selected characteristics of middle-aged South Australian women with (cases) and without (controls) breast cancer diagnosed in the previous 5 years

	Total	Cases (*n* = 80)	Controls (*n* = 235)	*p*-value*
Age last birthday (years)				
Mean	56.4	55.5	56.7	0.128
SD	6.5	6.5	6.6	
Median	57	57	57	0.118
IQR	51–61	51–61	52–62	
*[Missing]*	*[0]*	*[0]*	*[0]*	
Born in Australia – n (%) *[Missing]*	240 (75) *[0]*	60 (75) *[0]*	180 (77) *[0]*	0.772
Health status – n (%)				
Very good	133 (42)	20 (25)	113 (48)	< 0.001 (Cramer’s V = 0.31)
Good	126 (40)	31 (39)	95 (40)	
Moderate-fair	52 (17)	28 (35)	24 (10)	
Bad	4 (1)	1 (1)	3 (1)	
*[Missing]*	*[0]*	*[0]*	*[0]*	
Health status binary – n (%)				
Better	259 (82)	51 (64)	208 (89)	**< 0.001**
Poorer *[Missing]*	56 (18) *[0]*	29 (36) *[0]*	27 (11) *[0]*	(OR = 4.38)
BMI at each life period – median (IQR) *[Missing]*				
18–30	21.8 (20.1–24.0) *[6]*	22.2 (20.7–24.2) *[5]*	21.6 (20.0–23.8) *[1]*	0.118
31–40	23.4 (21.8–26.2) *[14]*	23.8 (23.3–27.1) *[6]*	23.0 (20.6–25.7) *[7]*	**0.014**
41–50	24.6 (21.6–28.4) *[14]*	25.7 (22.8–29.0 *[8]*	24.2 (21.3–28.1) *[6]*	**0.031**
51–60	25.3 (22.1–29.4) *[11]*	25.4 (23.4–29.8) *[6]*	25.3 (21.9–29.4) *[5]*	0.236
61–70	24.7 (21.5–29.6) *[6]*	26.5 (23.5–29.2) *[3]*	24.5 (21.2–30.1) *[3]*	0.280
Obesity (BMI ≥30) at each life period (years) – n (%) *[Missing]*				
18–30	13 (4) *[6]*	4 (5) *[5]*	9 (4) *[1]*	0.526
31–40	30 (10) *[14]*	13 (18) *[6]*	17 (8) *[8]*	**0.023**
41–50	46 (15) *[14]*	12 (17) *[8]*	34 (15) *[6]*	0.709
51–60	55 (24) *[11]*	12 (24) *[6]*	43 (24) *[5]*	1.000
61–70	21 (25) *[6]*	3 (20) *[3]*	18 (26) *[3]*	0.751
Ever smoked cigarettes – n (%) *[Missing]*	117 (37) *[0]*	31 (39) *[0]*	86 (37) *[0]*	0.731
Ever consumed alcohol – n (%) *[Missing]*	308 (98) *[0]*	79 (99) *[0]*	229 (97) *[0]*	0.495
Age first consumed alcohol (years)				
Mean	16.5	16.8	16.4	0.376
SD	3.0	3.3	2.8	
Median	16	16	16	0.996
IQR	15–18	15–18	15–18	
*[Missing]*	*[0]*	*[0]*	*[0]*	
Ever *regularly* consumed alcohol – n (%) *[Missing]*	265 (86) *[0]*	67 (86) *[0]*	198 (86) *[0]*	0.900
Age when started regular consumption (years) – n (%)				
12–17	23 (9)	2 (3)	21 (11)	**0.032** (Cramer’s V = 0.21)
18–30	192 (72)	53 (79)	139 (70)	
31–40	27 (10)	3 (4)	24 (10)	
41–50	13 (5)	5 (7)	8 (4)	
51–60	9 (3)	3 (4)	6 (3)	
61–70	1 (0)	1 (1)	0 (0)	
*[Missing]*	*[0]*	*[0]*	*[0]*	

*Difference between cases and controls using Chi-Square, Mann-Whitney, t-test as appropriate and Fisher’s 2-sided exact used in the case of small cell counts

Significant results (*p* < 0.05) in bold

**Fig. 2:**
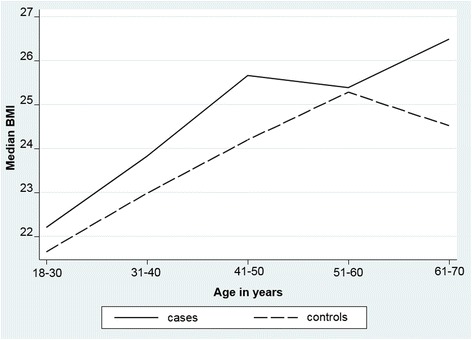
Body Mass Index (BMI) in cases and controls across 10-year life periods

For each 10-year life period relevant to the participants, data were collected on weight, alcohol consumption pattern, experience of stressful life events and activity level and smoking status. Based on their age when they completed the retrospective survey, 315 participants provided data for the ages 18 to 30 years, 31 to 40 years and 41 to 50 years; 245 participants provided data for the age 51 to 60 years; and 90 participants provided data for the age 61 to 70 years. In bivariate analyses, only weight and alcohol consumption differed significantly across age groups. The time trend for BMI (see Fig. 2) demonstrates higher mean BMI in cases for each age period. The difference reached significance when the women were aged 31 to 40 years (median 23. 8 versus 23.3, Mann-Whitney z = − 2.469, *p* = 0.014) and 41 to 50 years (median 25.7 versus 24.2, Mann-Whitney z = − 2.62, *p* = 0.031). Although the difference was greatest for those aged 61 to 70 years, it was not possible to demonstrate significance due to the relatively small numbers of cases and controls in this age group.

**Fig. 3:**
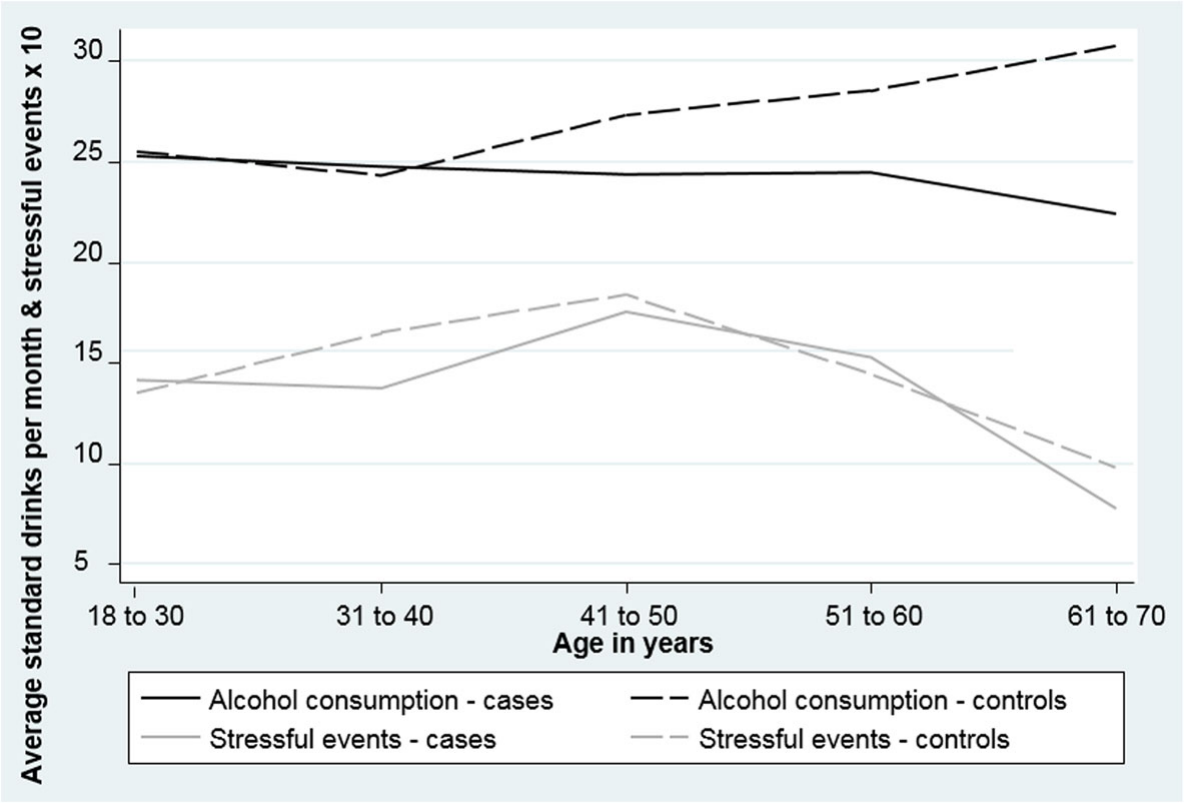
Standard drinks per month and number of stressful events* during 10 year life period in cases and controls. *Number of stressful events is multiplied by 10 to improve visibility in the graph

The reported exposures to regular alcohol consumption and stressful life events over the years are presented in Fig. 3. In this sample, there were few significant correlations indicative of interplay between the exposure variables. In the study participants, monthly alcohol consumption was not correlated with stressful life events reported in any life period. The Mann-Whitney associated with number of standard drinks per month was significant only when the participants were aged between 31 and 40 years (*p* < 0.05 in both cases and controls).

In relation to the additional variables, there was no difference in smoking status between cases and controls, with smoking prevalence declining for each life period, from 43% when aged 31 to 40 years to negligible proportions in older age groups. Among 86 women reporting data for the 61 to 70 year life period, only two reported smoking. Low activity level was positively associated with BMI in both cases and controls when the participants were aged 41 to 50 years (p < 0.05), but only in controls between the ages of 41 and 70 years (*p* < 0.005).

Exploring the interplay of all factors, correlation matrices for cases and controls are presented in Table 3, with correlations more common in controls than cases. In cases, there was a moderate positive correlation between smoking and alcohol consumption when the women were 31–40 years and a weak to moderate positive correlation between BMI and stress when the women were 41–50 years. In controls, smoking was weakly correlated with stress at the ages of 18 to 30 years and 31 to 40 years. As with cases, smoking and alcohol were correlated (weakly to moderately) in the 31 to 40 year age group. Engagement in only light activity and BMI were positively correlated in controls but not in cases in the three age groups comprising 41 to 70 years. Smoking and BMI were weakly correlated when controls were aged 51 to 60 years.

**Table 3:** Spearman’s rank correlation matrices for cases and controls – BMI, stressful events, alcohol intake, activity level and smoking prevalence across all each time periods

	BMI	Stress	Alcohol	Activity	Smoking
18 to 30 years					
Cases					
BMI	1.000				
No. of stressful life events	− 0.113	1.000			
Standard drinks per month	0.019	0.006	1.000		
Light activity prevalence	− 0.078	0.214	− 0.109	1.000	
Smoking prevalence	0.000	0.003	0.204	0.137	1.000
Controls					
BMI	1.000				
No. of stressful life events	0.052	1.000			
Standard drinks per month	0.067	0.065	1.000		
Light activity prevalence	0.090	−0.022	0.159	1.000	
Smoking prevalence	−0.040	0.174*	0.143	− 0.144	1.000
31 to 40 years					
Cases					
BMI	1.000				
No. of stressful life events	0.055	1.000			
Standard drinks per month	0.003	−0.053	1.000		
Light activity prevalence	0.120	−0.031	−0.045	1.000	
Smoking prevalence	0.030	−0.022	0.367*	0.153	1.000
Controls					
BMI	1.000				
No. of stressful life events	0.123	1.000			
Standard drinks per month	−0.037	− 0.035	1.000		
Light activity prevalence	0.132	−0.031	−0.020	1.000	
Smoking prevalence	0.056	0.142*	0.215*	0.034	1.000
41 to 50 years					
Cases					
BMI	1.000				
No. of stressful life events	0.283*	1.000			
Standard drinks per month	− 0.254	− 0.001	1.000		
Light activity prevalence	0.239	0.133	−0.124	1.000	
Smoking prevalence	0.101	−0.214	−0.075	0.135	1.000
Controls					
BMI	1.000				
No. of stressful life events	0.210*	1.000			
Standard drinks per month	−0.049	0.077	1.000		
Light activity prevalence	0.179*	0.143*	0.009	1.000	
Smoking prevalence	0.070	0.105	0.055	0.012	1.000
51 to 60 years					
Cases					
BMI	1.000				
No. of stressful life events	0.022	1.000			
Standard drinks per month	−0.182	0.137	1.000		
Light activity prevalence	0.245	0.239	0.068	1.000	
Smoking prevalence	−0.132	− 0.143	− 0.182	− 0.121	1.000
Controls					
BMI	1.000				
No. of stressful life events	0.034	1.000			
Standard drinks per month	−0.107	−0.016	1.000		
Light activity prevalence	0.295*	0.009	− 0.063	1.000	
Smoking prevalence	0.152*	−0.104	− 0.001	0.099	1.000
61 to 70 years					
Cases					
BMI	1.000				
No. of stressful life events	0.034	1.000			
Standard drinks per month	−0.377	0.126	1.00		
Light activity prevalence	0.024	−0.052	0.447	1.000	
Smoking prevalence**	–	–	–	–	–
Controls					
BMI	1.000				
No. of stressful life events	−0.010	1.000			
Standard drinks per month	−0.150	−0.021	1.000		
Light activity prevalence	0.322*	0.097	− 0.211	1.000	
Smoking prevalence**	–	–	–	–	–

*p < 0.05

**smoking omitted due to low cell counts

After no interactions between obesity and alcohol were identified, we built multivariate binomial models to identify any independent predictors of case status in each life period (as presented in Table 4). The only independent predictor was obesity when women were aged 31 to 40 years, when the odds of obesity in cases was 3.5 times that of controls and the absolute risk difference was 25%. Obesity was not important in any other life period and alcohol intake, stressful life events, smoking and activity level did not predict case status.

**Table 4:** Multivariate models predicting case-control status at each reported age group

Model*	Odds Ratio (95% CI)	Risk difference (95% CI)	*p*-value*
Aged 18 to 30 years (*n* = 170):			
Above median standard drinks per month (> 19)	0.83 (0.41–1.66)	− 0.04 (− 0.17–0.09)	0.513
Obese (BMI ≥ 30)	0.69 (0.07–6.28)	− 0.05 (− 0.36–0.27)	0.738
Above median number stressful life events (> 1)	0.78 (0.37–1.63)	−0.03 (− 0.06–0.10)	0.508
Smoking	0.73 (0.36–1.49)	−0.06 (− 0.20–0.07)	0.382
Low activity level	0.84 (0.35–2.06)	−0.05 (− 0.20–0.11)	0.708
Aged 31 to 40 years (*n* = 205):			
Above median standard drinks per month (> 16)	0.58 (0.28–1.16)	−0.09 (− 0.20–0.02)	0.123
Obese (BMI ≥ 30)	3.47 (1.28–9.42)	0.25 (0.03–0.47)	**0.015**
Above median number stressful life events (> 1)	0.76 (0.38–1.50)	−0.03 (− 0.14–0.08)	0.425
Smoking	0.82 (0.35–1.91)	−0.03 (− 0.17–0.10)	0.652
Low activity level	0.68 (0.31–1.50)	−0.06 (− 0.18–0.05)	0.341
Aged 41 to 50 years (*n* = 225):			
Above median standard drinks per month (> 16)	0.81 (0.42–1.53)	−0.03 (− 0.14–0.08)	0.511
Obese (BMI ≥ 30)	1.11 (0.46–2.65)	0.02 (− 0.15–0.18)	0.822
Above median number stressful life events (> 2)	0.97 (0.52–1.83)	0.00 (−0.11–0.11)	0.932
Smoking	0.59 (0.22–1.58)	−0.10 (− 0.30–0.11)	0.291
Low activity level	1.14 (0.58–2.24)	0.02 (− 0.10–0.14)	0.711
Aged 51 to 60 years (*n* = 163):			
Above median standard drinks per month (> 16)	0.81 (0.37–1.81)	− 0.04 (− 0.16–0.08)	0.614
Obese (BMI ≥ 30)	0.56 (0.19–1.66)	−0.07 (− 0.21–0.07)	0.293
Above median number stressful life events (> 1)	1.28 (0.57–2.85)	0.03 (− 0.09–0.15)	0.552
Smoking	0.54 (0.17–13.61)	0.07 (−0.16–0.30)	0.697
Low activity level	1.72 (0.70–4.32)	0.07 (− 0.07–0.22)	0.239
Aged 61 to 70 years (*n* = 86):**			
Above median standard drinks per month (> 20)	0.28 (0.06–1.20)	−0.16 (− 0.33–0.02)	0.085
Obese (BMI ≥ 30)	0.73 (0.10–5.41)	−0.04 (− 0.25–0.18)	0.760
Above median number stressful life events (> 0)	0.98 (0.24–4.04)	−0.02 (− 0.18–0.14)	0.980
Low activity level	0.67 (0.10–4.44)	−0.01 (− 0.19 – − 0.19)	0.679

*Log binomial models used

**smoking omitted due to low cell counts (2 smokers only)

Significant results (*p* < 0.05) in bold

Finally, we undertook a multilevel logistic regression accounting for repeated measures (see Table 5). Multicollinearity was identified between BMI and stress and BMI and activity level. Since these factors were not significant in any of the above analyses, they were omitted from the model. Model fit was evaluated by comparing different models using Akaike’s information criterion (AIC) and Schwarz’s Bayesian information criterion (BIC). Interaction between obesity and alcohol was also tested in the regression; however, this did not improve the model fit. The final model was fitted with the three remaining covariates: obesity, smoking status, alcohol consumption (average number of standard drinks per month) and time. The model fit was AIC = 265.84 and BIC = 294.78.

**Table 5:** Multilevel logistic regression, factors associated with case status

Case/control status	Odds Ratio (95% CI)	Coefficient (95% CI)	*p*-value
Obesity (BMI ≥ 30)	5.44 (0.20–147.37)	1.69 (−0.17–5.00)	0.314
Regularly smokes	0.62 (0.06–6.60)	−0.48 (− 2.84–1.89)	0.692
No. of standard drinks of alcohol per month	1.00 (0.96–1.03)	−0.04 (− 0.06–0.48)	0.823
Life period	0.75 (0.35–1.62)	−0.29 (− 1.06–0.48)	0.467

LR test of rho = 0: chibar2(01) = 723.98 Prob > = chibar2 = 0.000

A likelihood-ratio test comparing the model to ordinary logistic regression is highly significant for these data (*p* < 0.001). This suggests that a multilevel model was appropriate (Wald Chi^2^(4) = 1.54, Log likelihood = − 126.9180, Prob>Chi^2^ = 0.819), although no variable in the model was significantly associated with case status. While the odds of obesity in cases was almost 5.5 times than that of controls, this was not significant. Neither smoking nor alcohol consumption were important predictors of case status in this model, possibly reflecting the relatively small number of cases in each life period.

## Discussion

This study aimed to explore the inter-correlations of alcohol intake and overweight/obesity and their association with BC incidence in middle-aged women. The study also aimed to investigate the role of stress and various lifestyle factors in the relationships between alcohol, weight and BC. As an exploratory cross-sectional study, this study was not designed to establish causal relationships. Nonetheless, the investigation provides ecological evidence for moderate to strong correlations between population increases in obesity and alcohol consumption and BC incidence. Stress was not correlated with BC but was strongly correlated with alcohol consumption (positively) and obesity (negatively) in the ecological analysis. The case-control study demonstrated consistently higher BMI in cases relative to controls over time plus differing relationships between various lifestyle factors (stress, alcohol consumption, activity level and smoking status) in cases and controls, obesity emerging as a potentially important factor in BC development, but only among one age cohort; middle-aged women.

Somewhat supporting other evidence for a link between obesity and BC risk [66, 67], BMI tended to be higher in cases over time relative to controls, although only significantly so in the 31–40 and 40–51 year life periods. Consistent with previous research, the ecological analysis identified a strong correlation between BC and alcohol consumption, and a moderate correlation between obesity and BC incidence [1, 10–12, 68] but did not suggest any relationship between obesity and alcohol or a role for ‘overweight’ (as opposed to obesity) in BC development. In the case control analysis, the proportion of participants meeting the criterion for obese (i.e. BMI ≥ 30) did not differ according to case status; however, as mentioned, BMI was consistently higher in cases over time relative to controls. In the multivariate binary regressions, obesity when aged between 31 and 40 years was the single independent risk factor for BC. While the exact pathway to BC is still unknown, [69] the temporality between the exposure to obesity at earlier ages and later development of BC may be important. In the same age group (31 to 40 years), monthly alcohol intake and smoking were also more correlated in cases than in controls. Although not significant in the mixed multilevel regression model, obesity did demonstrate a large OR. While the failure to reach significance may reflect the size of our sample, it is also possible that the importance of obesity is specific to one life period (i.e. 31 to 40 years). This may have been masked in the combined random-effects model, which included time as a covariate. Collectively, our results suggest that obesity among women in their thirties may have independent effects on BC risk.

Contrary to the evidence for a link between alcohol consumption and BC risk [10, 70, 71], and the correlation seen in the ecological analysis, the case-control analysis found that monthly alcohol consumption was not associated with case status in any reported life period. Although it is possible that alcohol intake may have been inaccurately reported by participants, it is also possible that the other factors might modify the alcohol-BC relationship. Some studies that have investigated the effects of obesity and alcohol consumption on BC risk suggest an effect of alcohol on BC incidence among women with higher BMI. For example, in a prospective cohort study of early-stage BC survivors [72] regular drinking (3–4 standard drinks or more per week) was associated with increased risk of BC recurrence and BC death, and the observed associations were stronger among obese/overweight women. In contrast, Shin et al. [73] found that higher intake of alcohol was associated with an increase in overall BC risk only among women with BMI ≤25 kg/m^2^. According to the authors, it is likely that alcohol and obesity share common biological mechanisms in breast carcinogenesis through circulating sex hormone levels. The mixed results of our analyses confirm that further investigation into the interaction between these factors is warranted.

Stressful life events and accompanying psychological responses could increase BC risk by prompting behaviours implicated in the etiology of BC. Current research on the relationship between stressful life events and BC is inconclusive; Eskelinen and Ollonen [74]) reported that stress is a risk factor whilst other studies have not identified this link [75, 76]. In a cohort study of over 10,000 women it was found that stressful life events predicted BC risk independently of alcohol use, BMI and other lifestyle factors [36]. The ecological analysis did not provide evidence for a relationship between BC and stress, although we identified a strong pairwise relationship between *alcohol* and stress. This finding is consistent with evidence that chronic psychological stress is a contributing factor to patterns of harmful alcohol consumption [29]. Although a mediating relationship between stress, alcohol consumption and BC is plausible, alcohol consumption was not associated directly with stress in the case control analysis, and the reported number of stressful life events did not predict BC case status.

A negative correlation between obesity and stress was found in the ecological analysis, contradicting evidence that chronic stress is associated with over-eating and resulting weight gain [37]. In the case-control study, BMI was correlated with stress in case participants at the age of 41 to 50 years but not at any other time. Interestingly, low activity levels were correlated with BMI in controls but not in cases. It is important to note that the population level data measured subjective stress state using the K10 instrument, while number of external stressful events was measured in the case-control study participants. Differences in stress response – rather than stressful event occurrence per se – may be more important in the stress-BC relationship. Further research on response to stress and its relationship with BC development is warranted.

### Limitations and recommendations

As with all studies of population level data, our ecological analysis could be subject to the ‘ecological fallacy’, in that what is true at a population level may not necessarily be true on an individual level. Caution should also be taken in generalising our interpretations given that the data were not collected to answer our specific research questions. The later introduction of some of the data collection sets also indicates need for some caution in interpretation. Assuming ongoing consistency in data collection methods, such analyses could potentially prove to be valuable in monitoring population trends into the future.

The information collected from cases and controls was both self-reported and retrospective, which could be considered as a limitation of the study. Evidence indicates that women tend to underestimate their weight [77] and therefore relying on self-reported weight is likely to have provided less reliable information than objective measurements of BMI over time. Whether participants provided retrospective information for the older age groups was dependent on what age the participant was when they completed the survey and therefore data for the older age groups were fewer in number. This may account for the failure to reach significance between BMI and case status even though the pattern of higher BMI in cases persisted across age groups. The retrospective nature of the information also raises the potential for recall bias, as participants’ estimates of their alcohol consumption over several decades may not have been reliable. Although differential recall cannot be ruled out, the similarity in patterns of responses between cases and controls (as presented in Table 2) does not provide evidence for this. Thus, the comparison of responses from the two groups is likely to be valid despite the possibility of recall bias. As previously noted, the data collected in both parts of this study are cross-sectional in nature. Thus, it is not possible to demonstrate cause and effect and further studies are required to confirm the direction of any relationships identified.

Finally, while the power calculation for overall sample size was exceeded, small numbers in some subgroups occurred due to lower than expected prevalence of some of the exposure categories. For instance, the number of smokers in older age groups for both cases and controls was very low, which may have affected the validity of central tendency. Smoking, which is frequently associated with both stress [78] and alcohol consumption [79, 80] did not always correlate with either of these variables in matrix Spearman’s correlations in both cases or controls. It is possible that some of our multivariate modelling was also impacted by small numbers of cases in each category. A larger, prospective study of the interplay between alcohol, weight, stress and other life style factors and their complex relationships with BC development could provide urgently required information on which to base prevention strategies.

## Conclusion

This study provides ecological evidence for moderate to strong correlations between population increases in both obesity and alcohol consumption and BC incidence. The case-control study results suggest an association between obesity in women in their thirties and the later development of breast cancer. There were different stress response patterns between cases and controls. Although further study of these patterns and the complex connections between various other lifestyle factors is required, our findings support prevention efforts that target obesity in younger women and, potentially, lifelong alcohol consumption to reduce BC risk in middle-aged women.

## Abbreviations

BC: Breast cancer
BMI: Body mass index
LTE-Q: List of threatening experiences questionnaire

## References

[1] Colditz GA, Bohlke K (2014). Priority for the primary prevention of breast cancer. CA Cancer J Clin.

[2] Ory MG, Anderson LA, Friedman DB, Pulczinski JC, Eugene N, Satariano WA (2014). Cancer prevention among adults aged 45–64 years: setting the stage. Am J Prev Med.

[3] Australian Institute of Health and Welfare (2012). Breast cancer in Australia: an overview. Cancer series no.

[4] Australian Bureau of Statistics (2009). National Health Survey: summary of results, 2007–2008.

[5] Australian Bureau of Statistics (2013). Profiles of health, Australia, 2011–13.

[6] Sassi F (2015). Tackling harmful alcohol use: economics and public health policy.

[7] Watling H (2014). Media release: middle-aged tipplers: over 45s drink more frequently than young women.

[8] Fan AZ, Russell M, Dorn J, Freudenheim JL, Nochajski T, Hovey K, Trevisan M (2006). Lifetime alcohol drinking pattern is related to the prevalence of metabolic syndrome. The western New York health study (WNYHS). Eur J Epidemiol.

[9] World Health Organization, International Agency for Research on Cancer (1988). Alcohol drinking: summary of data reported and evaluation. IARC monographs on the the evaluation of carcinogenic risks to humans.

[10] Scoccianti C, Lauby-Secretan B, Bello P-Y, Chajes V, Romieu I (2014). Female breast cancer and alcohol consumption: a review of the literature. Am J Prev Med.

[11] Allen NE, Beral V, Casabonne D, Kan SW, Reeves GK, Brown A, Green J (2009). Moderate alcohol intake and Cancer incidence in women. J Natl Cancer Inst.

[12] Chen WY, Rosner B, Hankinson SE, Colditz GA, Willett WC (2011). Moderate alcohol consumption during adult life, drinking patterns, and breast cancer risk. JAMA.

[13] Singletary KWG, Susan M (2001). Alcohol and breast cancer: review of epidemiologic and experimental evidence and potential mechanisms. JAMA.

[14] Seitz HK, Pelucchi C, Bagnardi V, La Vecchia C (2012). Epidemiology and pathophysiology of alcohol and breast cancer: update 2012. Alcohol Alcohol.

[15] Colditz GA, Bohlke K (2014). Priorities for the primary prevention of breast cancer. CA Cancer J Clin.

[16] Kulie T, Slattengren A, Redmer J, Counts H, Eglash A, Schrager S (2011). Obesity and Women’s health: an evidence-based review. J Am Board Fam Me.

[17] Huang C-J, Zourdos MC, Jo E, Ormsbee MJ. Influence of Physical Activity and Nutrition on Obesity-Related Immune Function. Sci World J. 2013;2013:12. Article ID 752071. 10.1155/2013/752071. 10.1155/2013/752071 PMC3842061 24324381

[18] Calle EE, Kaaks R (2004). Overweight, obesity and cancer: epidemiological evidence and proposed mechanisms. Nature Rev Cancer.

[19] Kerr WC, Stockwell T (2012). Understanding standard drinks and drinking guidelines. Drug Alcohol Rev.

[20] Gazdzinski S, Durazzo TC, Watson RR, Preedy VR, Zibadi S (2013). Alcohol use and abuse: effects on body weight and body composition. Alcohol, nutrition, and health consequences.

[21] Traversy G, Chaput JP (2015). Alcohol consumption and obesity: an update. Curr Obes Rep.

[22] National Health and Medical Research Council (2013). Dietary guidelines for Australian adults.

[23] Australian Chronic Disease Prevention Alliance (2008). Alcohol and chronic disease prevention position statement.

[24] French MT, Norton EC, Fang H, Maclean JC (2010). Alcohol consumption and body weight. Health Econ.

[25] Santos R, Aires L, Santos P, Ribeiro JC, Mota J (2008). Prevalence of overweight and obesity in a Portuguese sample of adults: results from the Azorean physical activity and health study. Am J Hum Biol.

[26] Schröder H, Morales-Molina JA, Bermejo S, Barral D, Mándoli ES, Grau M, Guxens M, Jaime Gil E, Álvarez MD, Marrugat J (2007). Relationship of abdominal obesity with alcohol consumption at population scale. Eur J Nutr.

[27] Sung K-C, Kim SH, Reaven GM (2007). Relationship among alcohol, body weight, and cardiovascular risk factors in 27,030 Korean men. Diabetes Care.

[28] Gearhardt AN, Corbin WR (2009). Body mass index and alcohol consumption: family history of alcoholism as a moderator. Psychol Addict Behav.

[29] Hermansen K, Jørgensen K, Schmidt E, Tjønneland A, Tolstrup J, Grønbaek M (2007). Alcohol and lifestyle diseases. Ugeskr Laeger.

[30] Duvigneaud N, Wijndaele K, Matton L, Philippaerts R, Lefevre J, Thomis M, Delecluse C, Duquet W (2007). Dietary factors associated with obesity indicators and level of sports participation in Flemish adults: a cross-sectional study. Nutr J.

[31] Suter PM, Tremblay A (2005). Is alcohol consumption a risk factor for weight gain and obesity?. Crit Rev Clin Lab Sci.

[32] Dumalaon-Canaria JA, Hutchinson AD, Prichard I, Wilson C (2014). What causes breast cancer? A systematic review of causal attributions among breast cancer survivors and how these compare to expert-endorsed risk factors. Cancer Causes Control.

[33] Ferrucci LM, Cartmel B, Turkman YE, Murphy ME, Smith T, Stein KD, McCorkle R (2011). Causal attribution among cancer survivors of the 10 most common cancers. J Psychosoc Oncol.

[34] Panjari M, Davis SR, Fradkin P, Bell RJ (2012). Breast cancer survivors' beliefs about the causes of breast cancer. Psycho-Oncology.

[35] Kruk J (2012). Self-reported psychological stress and the risk of breast cancer: a case-control study. Int J Biol Stress.

[36] Lillberg K, Verkasalo PK, Kaprio J, Teppo L, Helenius H, Koskenvuo M (2003). Stressful life events and risk of breast Cancer in 10,808 women: a cohort study. Am J Epidemiol.

[37] National Cancer Institute Fact Sheet: Psychological stress and cancer. 2012. http://www.cancer.gov/cancertopics/factsheet/Risk/stress. Accessed 26 Mar 2018.

[38] Foss B, Dyrstad SM (2011). Stress in obesity: cause or consequence?. Med Hypotheses.

[39] Keyes KM, Hatzenbuehler ML, Hasin DS (2011). Stressful life experiences, alcohol consumption, and alcohol use disorders: the epidemiologic evidence for four main types of stressors. Psychopharmacology.

[40] Australian Cancer Incidence and Mortality (ACIM) books: Breast cancer. https://www.aihw.gov.au/reports/cancer/acim-books/contents/acim-books.

[41] National Heart Foundation of Australia (1980). Risk factor prevalence study.

[42] National Heart Foundation of Australia, University TAN (1983). Risk factor prevalence study: Australian data archive.

[43] National Heart Foundation of Australia (1989). The risk factor prevalence survey: Australian data archive.

[44] Australian Institute of Health and Welfare (1998). National Drug Strategy Household Survey, 1998.

[45] Vuksa P, Kelly J (1995). National Drug Strategy Household Survey, 1995.

[46] Australian Institute of Health and Welfare & Commonwealth Department of Health and Ageing (2001). National Drug Strategy Household Survey, 2001.

[47] Australian Institute of Health and Welfare & Commonwealth Department of Health and Ageing (2005). National Drug Strategy Household Survey, 2004.

[48] Australian Institute of Health and Welfare (2009). National Drug strategy household survey, 2007.

[49] Australian Institute of Health and Welfare (2011). National Drug Strategy Household Survey, 2010.

[50] Australian Institute of Health and Welfare (2015). National Drug Strategy Household Survey, 2013.

[51] Australian Bureau of Statistics (2013). Australian health survey: updated results, 2011–2012.

[52] Australian Bureau of Statistics (1994). 1989–90 National Health Survey: lifestyle and health Australia.

[53] Barry VW, Baruth M, Beets MW, Durstine JL, Liu J, Blair SN (2014). Fitness vs. fatness on all-cause mortality: a meta-analysis. Prog Cardiovasc Dis.

[54] Australian Bureau of Statistics. National Health Survey: Summary of Results - 1995. Canberra: ABS; 1997

[55] Australian Bureau of Statistics (2006). National Health Survey: summary of results, 2004–05.

[56] Kessler RC, Andrews G, Colpe LJ, Hiripi E, Mroczek DK, S-LT N, Walters EE, Zaslavsky AM (2002). Short screening scales to monitor population prevalences and trends in non-specific psychological distress. Psychol Med.

[57] Australian Bureau of Statistics. In: Statistics ABo, editor. Quarterly population estimates (ERP) by sex and age [data]: Canberra, ABS; 2015.

[58] Australian Bureau of Statistics. National Health Survey: Summary of Results - 1995. Canberra: ABS; 1997.

[59] National Health and Medical Research Council (2007). National Statement on ethical conduct in human research.

[60] Skinner HA, Sheu WJ (1982). Reliability of alcohol use indices. The lifetime drinking history and the MAST. J Stud Alcohol.

[61] Vasunilashorn S (2013). Retrospective reports of weight change and inflammation in the US National Health and nutrition examination survey. J Obesity.

[62] Brown W, Ball K. Physical activity and energy balance: Quick reference guide for health professionals. Camberra: National Heart Foundation of Australia; 2007 Available from: https://www.heartfoundation.org.au/images/uploads/publications/physical-activity-and-energy-balance.pdf. Accessed 26 Mar 2018.

[63] Brugha TS, Cragg D (1990). The list of threatening experiences: the reliability and validity of a brief life events questionnaire. Acta Psychiatr Scand.

[64] Geliebter A, Krawitz E, Ungredda T, Peresechenski E, Giese SY (2015). Physiological and psychological changes following liposuction of large volumes of fat in overweight and obese women. J Diabetes Obes.

[65] Çokluk Ö, Kayri M (2011). The effects of methods of imputation for missing values on the validity and reliability of scales. Ed Sci Theor Prac.

[66] Brown KA, Simpson ER. The Link Between Obesity and Breast Cancer Risk: Epidemiological Evidence, Chapter 2 in Brown KA and Simpson ER Obesity and Breast Cancer: The Role of Dysregulated Estrogen Metabolism. Australia: Springer; 5–10

[67] Van den Brandt PA, Spiegelman D, Yaun S-S, Adami H-O, Beeson L, Folsom AR, Fraser G, Goldbohm RA, Graham S, Kushi L (2000). Pooled analysis of prospective cohort studies on height, weight, and breast cancer risk. Am J Epidemiol.

[68] Singletary KW, Gapstur SM (2001). Alcohol and breast cancer: review of epidemiologic and experimental evidence and potential mechanisms. JAMA.

[69] Brash D and Cairns J. The mysterious steps in carcinogenesis. Br J Cancer. 2009;101(3):379–80. 10.1038/sj.bjc.6605171 PMC2720245 19638985

[70] Brooks PJ, Zakhari S (2013). Moderate alcohol consumption and breast cancer in women: from epidemiology to mechanisms and interventions. Alcohol Clin Exp Res.

[71] Romieu I, Scoccianti C, Chajes V, de Batlle J, Biessy C, Dossus L, Baglietto L, Clavel-Chapelon F, Overvad K, Olsen A (2015). Alcohol intake and breast cancer in the European prospective investigation into cancer and nutrition. Int J Cancer.

[72] Kwan ML, Kushi LH, Weltzien E, Tam EK, Castillo A, Sweeney C, Caan BJ (2010). Alcohol consumption and breast cancer recurrence and survival among women with early-stage breast cancer: the life after cancer epidemiology study. J Clin Oncol.

[73] Shin A, Sandin S, Lof M, Margolis KL, Kim K, Couto E, Adami HO, Weiderpass E (2015). Alcohol consumption, body mass index and breast cancer risk by hormone receptor status: Women’Lifestyle and health study. BMC Cancer.

[74] Eskelinen M, Ollonen P (2010). Life stress due to losses and deficit in childhood and adolescence as breast cancer risk factor: a prospective case–control study in Kuopio, Finland. Anticancer Res.

[75] Achat H, Kawachi I, Byrne C, Hankinson S, Colditz G (2000). A prospective study of job strain and risk of breast cancer. Int J Epidemiol.

[76] Hartz AJ, He T (2013). Cohort study of risk factors for breast cancer in post menopausal women. Epidemiol Health.

[77] Engstrom JL, Paterson SA, Doherty A, Trabulsi M, Speer KL (2003). Accuracy of self-reported height and weight in women: an integrative review of the literature. J Midwifery Women’s Health.

[78] Kassel JD, Stroud LR, Paronis CA (2003). Smoking, stress, and negative affect: correlation, causation, and context across stages of smoking. Psychol Bull.

[79] Grant BF, Hasin DS, Chou SP, Stinson FS, Dawson DA (2004). Nicotine dependence and psychiatric disorders in the United States: results from the national epidemiologic survey on alcohol and relatedconditions. Arch Gen Psychiatry.

[80] Bobo JK, Husten C (2000). Sociocultural influences on smoking and drinking. Alcohol Res Health.

